# Characterization *In Vitro* and *In Vivo* of a Pandemic H1N1 Influenza Virus from a Fatal Case

**DOI:** 10.1371/journal.pone.0053515

**Published:** 2013-01-10

**Authors:** Ariel Rodriguez, Ana Falcon, Maria Teresa Cuevas, Francisco Pozo, Susana Guerra, Blanca García-Barreno, Pamela Martinez-Orellana, Pilar Pérez-Breña, Maria Montoya, Jose Antonio Melero, Manuel Pizarro, Juan Ortin, Inmaculada Casas, Amelia Nieto

**Affiliations:** 1 Centro Nacional de Biotecnología, C.S.I.C. Darwin 3, Cantoblanco, Madrid, Spain; 2 Ciber de Enfermedades Respiratorias, Mallorca, Illes Balears, Spain; 3 Centro Nacional de Microbiología, Instituto de Salud Carlos III, Majadahonda, Madrid, Spain; 4 Dpto. de Medicina Preventiva, Salud Pública y Microbiología, Universidad Autónoma de Madrid, Madrid, Spain; 5 Centre de Recerca en Sanitat Animal (CReSA), UAB-IRTA, Campus de la Universitat Autònoma de Barcelona, Bellaterra, Barcelona, Spain; 6 Institut de Recerca i Tecnologia Agroalimentarias (IRTA), Barcelona, Spain; 7 Servicio de Anatomia Patologica, Hospital Clínico Veterinario, Facultad de Veterinaria, Universidad Complutense, Madrid, Spain; University of Freiburg, Germany

## Abstract

Pandemic 2009 H1N1 (pH1N1) influenza viruses caused mild symptoms in most infected patients. However, a greater rate of severe disease was observed in healthy young adults and children without co-morbid conditions. Here we tested whether influenza strains displaying differential virulence could be present among circulating pH1N1 viruses. The biological properties and the genotype of viruses isolated from a patient showing mild disease (M) or from a fatal case (F), both without known co-morbid conditions were compared *in vitro* and *in vivo*. The F virus presented faster growth kinetics and stronger induction of cytokines than M virus in human alveolar lung epithelial cells. In the murine model *in vivo*, the F virus showed a stronger morbidity and mortality than M virus. Remarkably, a higher proportion of mice presenting infectious virus in the hearts, was found in F virus-infected animals. Altogether, the data indicate that strains of pH1N1 virus with enhanced pathogenicity circulated during the 2009 pandemic. In addition, examination of chemokine receptor 5 (*CCR5*) genotype, recently reported as involved in severe influenza virus disease, revealed that the F virus-infected patient was homozygous for the deleted form of *CCR5* receptor (*CCR5Δ*32).

## Introduction

Influenza A viruses are endemic in many wild avian species and can produce infections in mammals with varying morbidity and mortality rates. In humans they cause annual epidemics and occasional pandemics of respiratory disease with potentially fatal outcome. Since 1997, highly pathogenic avian H5N1 viruses have sporadically produced infections in humans but although this emerging virus presented a high mortality rate it showed poor transmissibility between humans and did not spread over human population [1]. By contrast, a new influenza A virus from H1N1 subtype, possessing high transmissibility but relatively low virulence, emerged in 2009 (pH1N1) rapidly spreading across the entire globe and causing the first pandemic of the 21st century [2], [3]. Infection with the new pandemic viruses produced mild symptoms in the majority of infected people but compared with the previous seasonal H1N1, they caused a greater rate of severe or complicated illness in healthy young adults and children [4], [5].

The co-morbid conditions were similar for both the pH1N1 and the previous seasonal influenza viruses and include chronic metabolic disease, primarily diabetes mellitus and renal disease, chronic lung and cardiac disease, immunosuppressive conditions and neoplasms [6]. In addition, obesity and pregnancy were associated to pH1N1 severe infections [7], [8], [9]. The pre-existing immune status and the existence of underlying chronic conditions definitely contributed to the patient outcome. However, the reasons why some pandemic H1N1 infected patients developed severe disease and even died, while others did not are still unknown, as some of the infected patients developing severe symptoms did not present obvious impairments in health condition.

To analyze possible virulence differences, we have compared two contemporary human pH1N1 viruses from two patients without known co-morbid conditions, one that became fatal while the other showed only mild respiratory disease. The results indicate that the virus isolated from the fatal case replicates faster, induces higher levels of cytokines in human alveolar lung epithelial cells and is more pathogenic in a murine model *in vivo*, compared with the virus obtained from the patient with mild disease. In addition, the deceased patient was homozygous for the *CCR5Δ32* allele, a rare genetic background found in less than 1% of the population [10], while the other patient was wild type for *CCR5* allele.

## Materials and Methods

### Ethics Statement

The National Influenza Center in Madrid (which belonged to the Instituto de Salud Carlos III) and other regional laboratories from different Spanish regions, constituted the ReLEG network included in the Spanish Influenza Surveillance System (SISS), which monitored the circulation of influenza viruses each influenza season as a part of the countrywide surveillance. This study has been developed within this activity, which was approved by the institutional review board at the Instituto de Salud Carlos III.

All the procedures that required the use of animals complied with Spanish and European legislation concerning vivisection and the use of genetically modified organisms, and the protocols were approved by the National Center for Biotechnology Ethics Committees on Animal Experimentation and the Consejo Superior de Investigaciones Científicas (CSIC) Bioethics Subcommittee. In particular, we follow the Guidelines included in the current Spanish legislation on protection for animals used in research and other scientific aims: RD 1201/2005, 10 October and the current European Union Directive 86/609/CEE, DOCE 12.12.86 (N.L358/1 to N.L358/28) on protection for animals used in experimentation and other scientific aims.

### Viruses

Two distinct influenza viruses named A/CastillaLaMancha/RR5661/2009 (M) and A/CastillaLaMancha/RR5911/2009 (F), were isolated at the National Influenza Centre (CNM, ISCIII) from respiratory samples sent by the Spanish Influenza Surveillance System for virological characterization. Isolations were made at the middle stage of the 2009 pandemic. Both viruses belong to Caucasian individuals. Virus M was detected in a 23 years old man and virus F was isolated from a 35 years old woman. Both viruses were isolated from bronchoalveolar lavages, collected in 3 ml virus transport medium (MEM, 200 U/ml penicillin, 200 µg/ml streptomycin, 200 U/ml mycostatin and 0,25% bovine albumin fraction V). Semi-confluent monolayers of MDCK cells were used for primary viral isolation. The monolayers were inoculated with 200 µl of homogenized samples and when the cytopathic effect was 75–100%, the cultures were harvested and the supernatants used for virus stock generation by inoculation of MDCK cells.

### Virion Purification and Viral Genome High-throughput Sequencing

For virus purification, culture supernatants of MDCK-infected cells were centrifuged for 10 min at 10,000 rpm and 4°C. The supernatants were sedimented through a sucrose step gradient (TNE; 50% and 33% in 50 mM Tris-HCl, 100 mM NaCl, 5 mM EDTA, pH 7.5) for 1 h at 40,000 rpm and 4°C in a SW41 rotor. The 50 to 33% interphase was collected, diluted in TNE buffer, and pelleted through a cushion of 33% sucrose in TNE for 1 h at 40,000 rpm and 4°C in a SW41 rotor. The isolation of total RNA from the pellet was carried out using RNAeasy isolation reagent (Quiagen) according to the manufacturer’s instructions. Appropriate amounts of each sample were analyzed by high-throughput sequencing as indicated below.

Library preparation was performed using the Illumina mRNA seq sample preparation kit (Illumina kit RS-100-0801) as previously described [11]. The quality of libraries was confirmed with the Agilent 2100 Bioanalyzer. Sequencing was performed on the Illumina Genome Analyzer IIx using Illumina v5 sequencing chemistry and a 36 cycle recipe. Base calling was performed using Illumina pipeline version 1.7.0 (within SCS 2.8). Reads were aligned versus the genome of influenza A/California/04/2009 virus by illumina’s ELAND algorithm. The most abundant nucleotide in each position was considered as the “consensus”. The algorithm MUSCLE [12] was used for aligning all pandemic segments (amino acid sequences).

### *In vitro* Infection

Cultured human lung alveolar epithelial cells (A549) from the American Type Culture Collection (ATCC) were infected at 10^−3^ PFU/cell (low moi) or 3 PFU/cell (high moi), after 1 h the non-bound virus was rinsed off with acid PBS (pH 5.3) and at different hours post-infection (hpi), cell supernatants were collected and used for virus titration by plaque assay.

### Cytokine Determination

The secretion of cytokines by human alveolar lung epithelial cells (A549) infected at 3 PFU/ml with different influenza viruses was measured 12 and 24 hpi in the culture supernatant using the Luminex 100 technology (Cytokine Human LINCOplex, Linco Research, Inc. St. Louis, MO, USA), following the manufacturer’s instructions. The following 15 mediators were tested: granulocyte-macrophage colony-stimulating factor (GM-CSF), interleukin-1α (IL-1α), IL-1β, IL-6, IL-8, IL-10, IL-15, alpha 2 interferon (IFNα), IFN-γ, interferon gamma-induced protein 10 (IP-10), monocyte chemotactic protein 1 (MCP-1), macrophage inflammatory protein 1β (MIP-1β), Regulated upon Activation, Normal T-cell Expressed, and Secreted (RANTES) and tumor necrosis factor α (TNF-α) and TNFβ.

### *In vivo* Infection

Female BALB/c AnNHsd mice (6–8 weeks old) were infected intranasally with 10^6^ PFU of either M or F influenza viruses or were mock infected. The animals were monitored daily for clinical signs and body weights. On days 2, 4, 7 and 14 dpi mice were euthanized and necropsied. Clinical specimens from lungs, kidneys, hearts and brains were homogenized in phosphate-buffered saline-0.3% bovine serum albumin in a Dounce homogenizer and used for determination of viral titers by plaque assay.

### Virus Titration

Tissue samples were homogenized (10% [wt/vol]) in PBS and debris was pelleted by centrifugation (2,000 *g*, 5 min). Virus titers of triplicate tissue samples or cell culture supernatants were determined by standard plaque assay on MDCK cells.

### Histopathology and Immunohistochemistry

Animal tissues were fixed in 10% formalin, embedded in paraffin, sliced into 5 µm-thick sections, and stained with hematoxylin and eosin (H&E) by conventional methods. To visualize influenza virus in animal lungs, a polyclonal nucleoprotein (NP) antibody [13] was diluted in Tris buffered saline (TBS) at 1∶5000, followed by incubation over night. Next, a biotinylated secondary goat anti-rabbit antibody (Vector, Burlingame, CA) was incubated for 30 min, followed by streptavidin horseradish peroxidase conjugate (Invitrogen, Carlsbad, CA). Specificity of staining was confirmed by omitting the primary antibody. Immunostaining was revealed with DAB Peroxidase Substrate kit 3, 3′-diaminobenzidine (Vector, Burlingame, CA). Slides were counterstained with hematoxylin, dehydrated, cleared in xylene, and coverslipped.

### *CCR5* Allele Determination

Genotyping was performed by standard methods described elsewhere [14]. Briefly, the RNA from bronchoalveolar lavages of patients, mice kidneys or cultured A549 cells, was amplified using previously reported primers surrounding the 32-bp deletion in the *CCR5* gene [15] for patient and A549 cells and 5′-CATTATACATGCAGTCCTC-3′ and 5′-GATGGCAAAGATGAGCCTAC-3′ primers for mice DNA.

## Results

To determine possible differences in virulence, we compared the biological properties of two distinct influenza viruses named mild (M) and fatal (F), both isolated from young patients without known health risk conditions. Virus M was detected in a patient that presented mild respiratory symptoms, whereas virus F was isolated from a patient that developed severe pneumonia, was hospitalized in the intensive care unit and died several days after admission.

### Genetic Characterization of M and F Viruses

**Figure 1:**
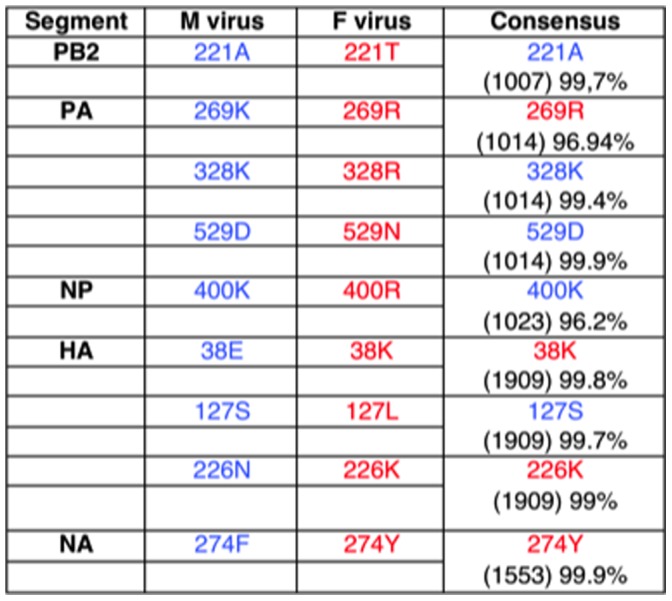
Amino acid differences between M and F viruses. Residues found in M or F viruses are represented in blue or red respectively. “Consensus” represents an amino acid sequence obtained using the influenza virus resource database from NCBI and including around one thousand 2009 pandemic viruses isolated in the time frame of one month before and after the isolation date of M and F viruses. Numbers in parenthesis represent the number of examined sequences, followed by the percentage of the corresponding amino acid present in these sequences.

Genetic characterization of M and F viruses was performed by ultrasequencing of purified virion RNAs obtained after two passages in MDCK cells. The consensus sequences obtained for the viral genomes represent more than 95% of the total sequence reads at each position. The two viruses differed in 29 nucleotides distributed over all the RNA segments, which produce 9 amino acids changes affecting PB2 (1 aa), PA (3 aa), NP (1 aa), HA (3 aa) and NA (1 aa). Three of these amino acid changes involve conservative substitutions of basic residues (aa 269 and 328 in PA and aa 400 in NP) (Fig. 1). The amino acid changes between M and F viruses involve single nucleotide variations within the triplets that encode the corresponding residue and could be considered sequence positions in the process of genetic drift. Therefore we examined the percentage of each nucleotide within these triplets and the results showed that the vast majority of sequences read at these positions represent the annotated amino acid (Table S1). To serve as a reference, a consensus amino acid sequence was obtained using the influenza virus resource database from NCBI and including around one thousand 2009 pandemic viruses isolated in the time frame of one month before and after the isolation date of M and F viruses. Residues that differ between these viruses are presented in Fig.1 for comparison and residues found in M or F viruses are represented in blue or red, respectively. Residues HA 38K, HA 226K and NA 274Y found in the F virus are also present in the consensus sequence, but residues HA 127L, PB2 221T and PA 529N were only detected in F virus and appeared as particularly interesting. To exclude their appearance in the virus stocks generated in MDCK cells, its presence in the original F clinical isolate was confirmed by sequencing. None of the changes in the PB2 and PA subunits had been previously associated with increased virulence [16], [17] nor they involve the PB1-interacting domains [18], [19], [20], [21], the PA endonuclease active site [22], [23], the PA proteolysis induction domain [24] or the PB2 cap-binding site [25]. The changes observed in the HA and NA glycoproteins have not been associated previously with high virulence in either H5N1 avian strains [26] or 2009 H1N1 pandemic viruses [27], [28]. On the other hand, none of the previously described pathogenic determinants were found in the M or F viral sequences.

### Biological Characterization of M and F Viruses in Human Alveolar Lung Epithelial Cells

**Figure 2:**
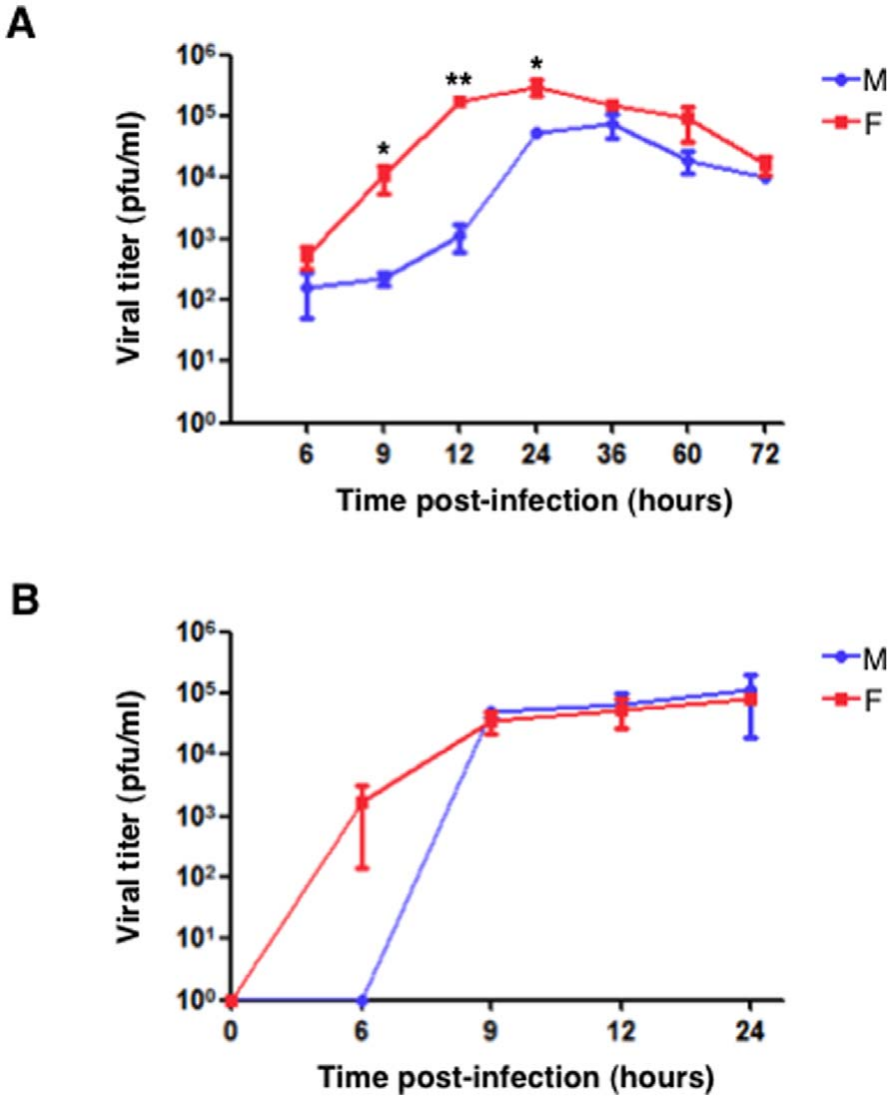
F virus replicates earlier than M virus. (A), Cultured A549 cells were infected at 10^−3^ PFU/cell with the M or F isolates of influenza virus. At the indicated hpi cell supernatants were collected and the virus titer determined by plaque assay in MDCK cells. Three independent experiments were carried out and a representative experiment is shown. Error bars indicate the standard deviation of the mean. (B), Cultured A549 cells were infected at 3 PFU/cell with the M or F isolates of influenza virus. At the indicated hpi cell supernatants were collected and the virus titer determined by plaque assay in MDCK cells. Three independent experiments were carried out and a representative experiment is shown. Error bars indicate the standard deviation of the mean. Student’s t-test was performed to determine the *P* value. *P<0.05, **P<0.01.

To analyze the properties of these viruses, cultures of human alveolar lung epithelial cells (A549) were infected with each virus at low multiplicity of infection and viral titers were determined at different hpi. A difference of more than 2 logs in the viral titers was observed shortly after infection (9–12 h). However, both viruses reached similar titers at later times (Fig. 2A). The viral replication kinetics were also examined for M and F viruses at high multiplicity of infection (Fig. 2B). The F progeny virus was detected as early as 6 hpi, whereas viral production was undetectable before 9 hpi in M virus-infected cells. Therefore, the F virus replicates faster than M virus both at low and high multiplicity of infection.

### Cytokines Induction of M and F Viruses in Cultured Cells

**Figure 3:**
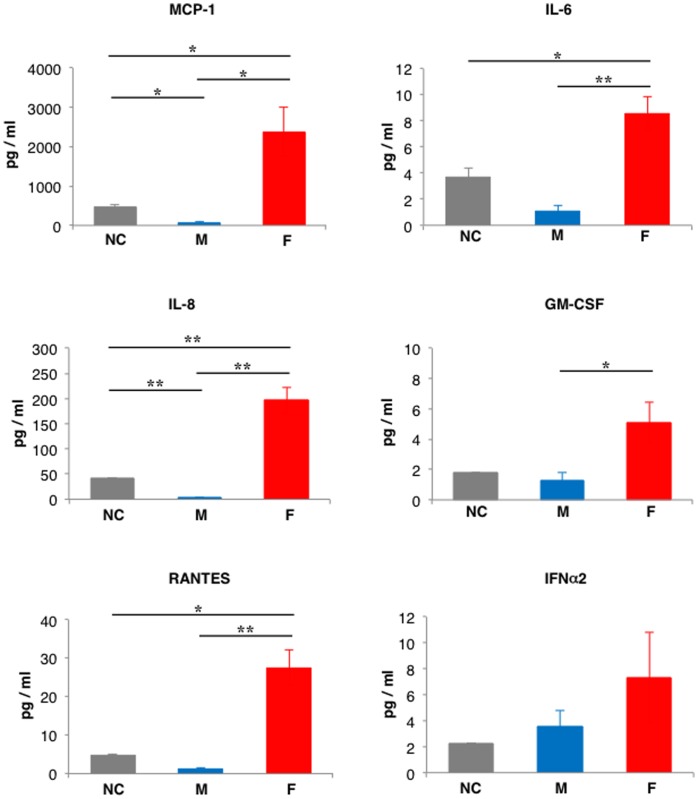
Profile of cytokines induction on A549 infected cells. Samples of A549 cells were infected in triplicate at 3 PFU/cell with the A/New Caledonia/20/99 (NC), the M or the F pandemic H1N1 isolates. At 12 hpi cell supernatants were taken and used to determine the concentration of the indicated cytokines using the Luminex 100 System. Error bars indicate the standard deviation of the mean. Student’s t-test was performed to determine the *P* value. *P<0.05, **P<0.01.

To determine the capacity of the M and F viruses to induce cytokines, their profiles were evaluated in infected human alveolar lung epithelial cells. For comparison, the cells were also infected with previous seasonal H1N1 virus, the A/New Caledonia/20/99 (NC) strain. Samples of A549 cells were infected in triplicate and in all cases the cytokine induction was evaluated at 12 and 24 hpi using the Luminex 100 System. At 12 hpi, F virus-infected cells showed increased accumulation of pro-inflammatory cytokines such as IL-6 (8-fold) or IL-8 (65-fold), chemokines such as RANTES (20-fold) as well as MCP-1 (371-fold), colony-stimulating factor GM-CSF (4-fold) and IFN-α (2-fold) as compared with M-infected cells (Fig. 3). These differences were also observed when comparing F virus-infected cells with those infected with the seasonal strain (NC), reinforcing the notion that the human alveolar lung epithelial cells displayed an enhanced cytokine response when infected with the F virus (Fig. 3). The differences between F and M-infected cells were dissipated at 24 hours post-infection (Fig. S1) suggesting that the cytokine induction occurred earlier in F virus- than in M virus-infected cells. These results are consistent with the faster replication of F virus in cell cultures. Other cytokines, such as IFN-γ, IL-1α, IL-1β, IL-10, MIP-1β, IL-15, IP-10, TNF-α and TNF-β, were also examined but their amounts were near or under the detection limit (data not shown). Overall, the earlier and stronger induction of the pro-inflammatory cytokines and chemokines observed in F virus-infected cells correlates with the higher degree of virulence of this strain in the patient.

### Characterization of M and F Infection in Mice

**Figure 4:**
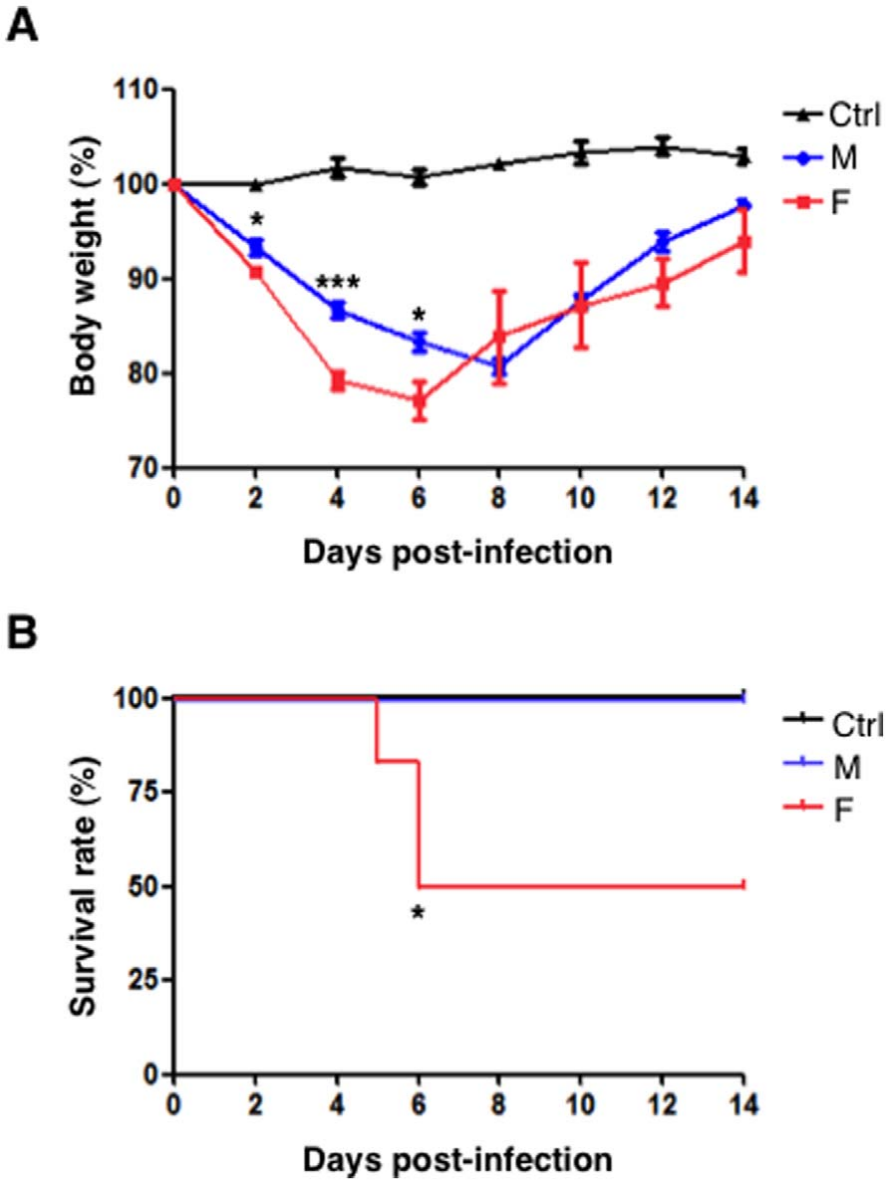
F virus is more pathogenic than M virus *in vivo*. Six mice were intranasally inoculated with 10^6^ PFU (50 µl) of either M or F influenza viruses or were mock infected with 50 µl of PBS. (A), Body weights were determined daily for 14 days and are depicted as the percentage of body weights at time of inoculation. Data show body weights mean of mice (n = 6). Error bar represent standard deviation. (B), Mice were monitored daily for survival for 14 days. Animals that lost 25% of its body weight were euthanized and counted as dead animals. For body weights, Student’s t-test was performed to determine the *P* value. *P<0.05, ***P<0.001. For survival, statistical significance was assessed by a Logrank (Mantel-Cox) Test. *P<0.05.

Since F virus replication was faster than M virus in cell cultures, we examined the *in vivo* relevance and the consequences of this difference in the murine model. Six BALB/c mice were intranasally infected with 10^6^ PFU of M or F viruses or were mock-infected. Survival and body weight were monitored daily for two weeks. No loss of body weight was observed in mock-infected mice whereas mice infected with M and F viruses had a peak of weight loss 5 to 7 days after inoculation. However, in the F virus-infected mice the weight loss was more pronounced and the recovery of body weight was slower (Fig. 4A). Importantly, 50% lethality on F virus-infected mice was observed in comparison with 100% survival on M virus- or mock-infected animals (Fig. 4B). In addition, clinical symptoms such as piloerection or general activity correlated with the morbidity (data not shown). Altogether these results confirm that F virus is more pathogenic than the M isolate in the murine model.

#### Virus Replication

**Figure 5:**
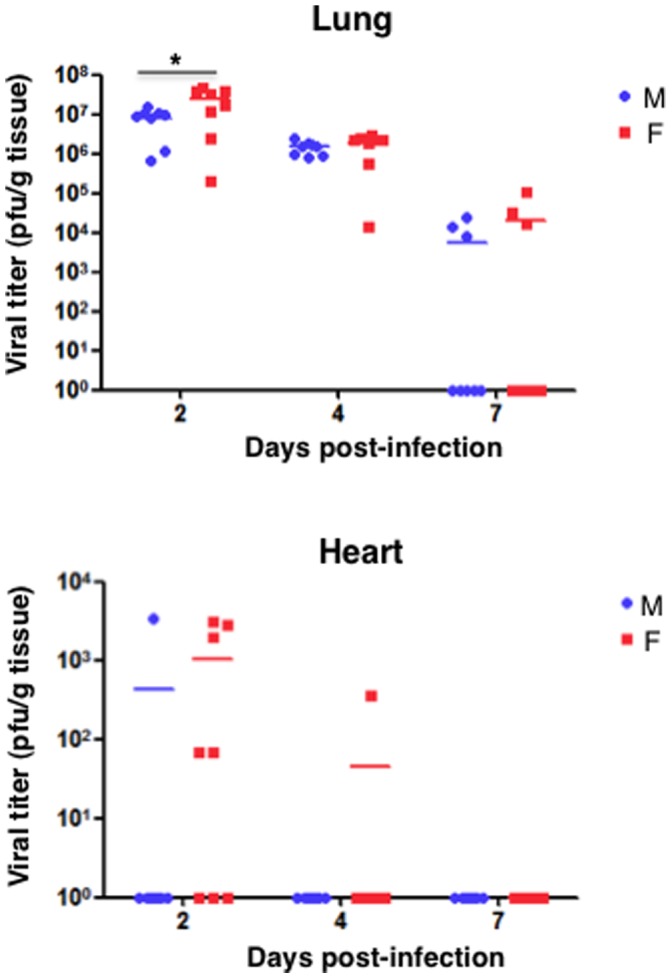
Virus titer in organs of M and F infected mice. Mice were intranasally inoculated with 10^6^ PFU (50µl) of either M or F influenza viruses. Eight mice from each group were euthanized on days 2, 4 and 7 post-infection for virus titration. Bars show mean of each group. Student’s t-test was performed to determine the statistical significance. *P<0.05.

Since influenza virus primarily infects the lungs of mice, lung samples of eight infected animals were used to determine the viral titers at different days post-infection (dpi) (Fig. 5, Lung). At two dpi virus titer reached its maximum value and was higher in F-infected mice. The presence of virus was reduced gradually in the lungs of both groups of infected animals and by 7 dpi still infectious virus was detectable, with higher viral titers in F-infected mice but this observation was not statiscally significant.

The detection of pH1N1 influenza virus in organs different from the lung, such as kidney and brain has been reported [29], [30], [31], [32] and heart dysfunction has been associated with pH1N1 influenza virus infection [33], [34], [35]. Therefore, we tested the possible presence of influenza virus in these organs. Infectious virus was not detected in the kidneys or brains but it was detected in the hearts (Fig. 5 Heart). Five of the eight F virus-infected animals presented infectious virus at 2 dpi and one of them presented infectious virus at 4 dpi. Infectious virus was found only in one of the M-infected animals at 2 dpi (Fig. 5).

#### Histopathology

**Figure 6:**
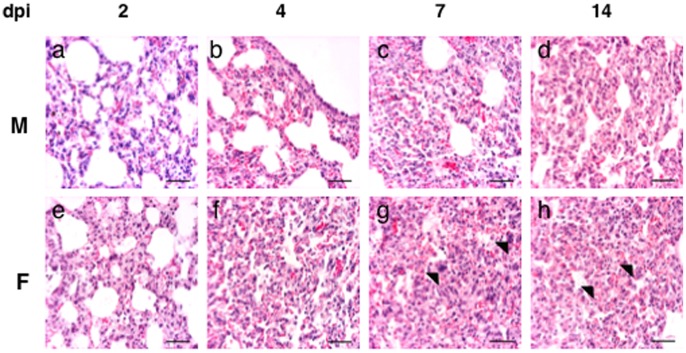
Histopathology of lungs of mice inoculated with M virus (a–d) or F virus (e–h). Lung samples of M and F virus-infected mice were used for histopathological H&E examination at the indicated days post-infection. ba r = 50 µm. Lung of mouse inoculated with M virus, a) 2 dpi. Mild congestion and diffuse lymphoid and phagocytic cells infiltrates in the interstitium. b) 4 dpi. Moderate thickened of interalveolar walls c) 7 dpi. Interstitial pneumonia with moderate proliferation of pneumocytes type II. d) 14 dpi. Interstitial pneumonia with thickened interalveolar walls. Lung of mouse inoculated with F virus, e) 2 dpi. Thickened interalveolar walls with consolidation of the pulmonary parenchima (interstitial pneumonia). f) 4 dpi. Moderate increase of interstitial macrophages. g) 7 dpi. Increase of macrophages and syncitial cells formation. h) 14 dpi. Severe hyperplasia of phagocytic cells in the interstitium. The arrows show some examples of the lesion indicated in each case.

**Figure 7:**
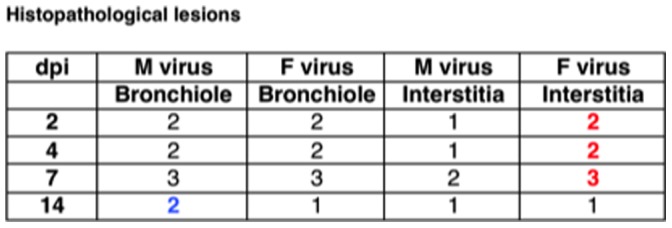
Histopathological signs of the lungs of the M and F-infected mice. Histopathological lesions (1: mild; 2: moderate; 3: severe). Numbers in blue (M virus) or red (F virus) highlight histopathological differences between both viruses.

In domestic animals influenza viruses produce bronchointerstitial pneumonia characterized by the appearance of both bronchiolar necrosis and diffuse alveolar damage [36]. Then, we investigated whether histopathological differences were present in the lungs of M and F virus-infected mice at three different days post-infection. Macroscopically, the lungs of the F mice displayed evident signs of necrosis while the lungs of the M mice displayed a moderate damage (data not shown). Microscopically, the lungs of both M and F-infected mice showed bronchiolar necrosis with bronchioles filled of cellular debris that became more severe with the progression of the infection. In addition, proliferation of macrophages and type II pneumocytes was observed, as a representative lesion of interstitial pneumonia together with vasculitis with mild interstitial reaction at late dpi (data not shown). However, F-infected mice showed bronchointerstitial pneumonia with more interstitial compound and early in the infection (2 dpi) in comparison to M- infected mice (7dpi) (Fig. 6). Moreover, in the F-infected lungs formation of syncitial cells by 7 dpi was evident and hyperplasia of phagocytic cells was observed at 14 dpi, both as representatives of inflammation. This prominent histopathological damage that distinguishes the M and F *in vivo* infection and is summarized in Fig. 7, correlates with the clear differences previously observed in the mortality rates in the mouse model (Fig. 4).

#### Immunohistochemistry

**Figure 8:**
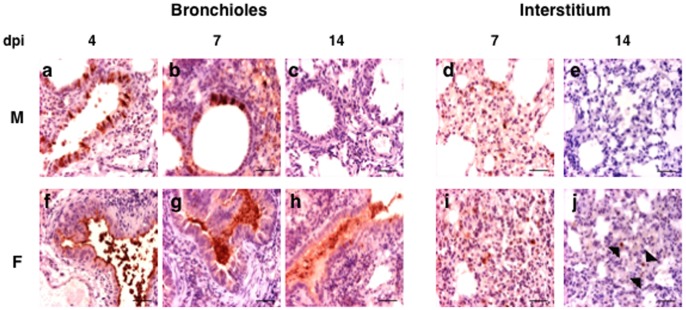
Immunostaining of lungs of mice inoculated with M virus (a–e) or F virus (f–j). Lung samples of M and F virus-infected mice were used for immunostaining with anti-influenza nucleoprotein antibody, bar = 50 µm. Lung of mouse inoculated with M virus, a) 4 dpi. Moderate immunoreaction in the epithelial cells of the bronchiole, b) 7 dpi. Mild to moderate immunoreaction in the epithelial cells of the bronchiole and in the macrophages of the interstititum, c) 14 dpi. Negative immunorection in all the pulmonary structures, d) 7 dpi. Moderate immunoreaction in the phagocytic cells of the pulmonary interstitium, e) 14 dpi. Negative immunorection in the interstitium. Lung of mouse inoculated with F virus, f) 4 dpi. Moderate immunoreactions in the apical pole of epithelial cells of the bronchiole, g) 7 dpi. Moderate immunoreaction in the necrotic and phagocytic cells desquamates into bronchiolar lumen, h) 14 dpi. Mild immunoreaction in the necrotic debris into bronchiolar lumen. Moderate immunoreaction in the pulmonary interstitium, i) 7 dpi. Moderate immunoreaction in the phagocytic cells of the interstitium with consolidation of the lung (Interstitial lung disease), j) 14 dpi. Mild immunoreaction in the phagocytic cells of the interstitium. The arrows show some influenza virus infected cells.

**Figure 9:**
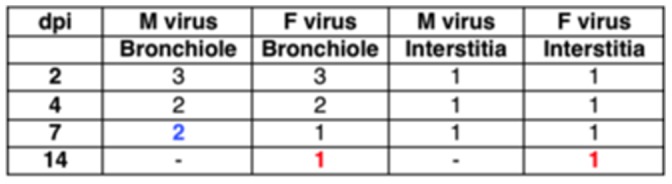
Immunohistochemistry of the lungs of the M and F-infected mice. Positive reaction to viral antigen in the bronquiole (epithelium and/or desquamate cells) and interstitia (pneumocytes and/or macrophages) at different days of inoculation. (-: absence; 1: mild; 2: moderate; 3: strong). Numbers in blue (M virus) or red (F virus) highlight immunohistochemistry differences between both viruses.

Next, immunohistochemistry analysis was performed to detect the presence of NP viral protein in the lung cells of M and F virus-infected mice at different days post-infection. Animals inoculated with either virus showed very strong immunostaining reaction in bronchiolar epithelial cells at 2 dpi and in the interstitial cells at 2–4 dpi (data not shown). In M virus-infected mice the bronchiolar reaction was progressively reduced to become negative at 14 dpi (Fig. 8 a–c). The interstitial reaction, in macrophages and/or type II pneumocytes, was moderate in isolated cells at 7 days and negative after 14 dpi (Fig. 8 d,e). Mice inoculated with F virus showed at 4 and 7 dpi a moderate to strong antigen positive bronchiolar reaction, (Fig. 8 f–h) and the interstitial reaction was moderate in isolated cells between 7 and 14 dpi (Fig. 8 i,j). The most striking differences between the immunohistochemical features in the two animal groups was that at 14 dpi antigen-positive bronchiolar desquamated cells were still detected and a mild reaction appeared in isolated interstitial macrophages in the F-inoculated animals. A summary of NP antigen detection in the lungs of the infected mice is presented in Fig. 9. Although no viral titer was detected in lungs of any mice by 14 dpi, discrete viral antigen positive cells were present in lungs of F virus but not M virus-infected mice, suggesting a slower kinetics of F virus clarification.

### Determination of Chemokine Receptor 5Δ32 Allele in M and F- infected Patients

**Figure 10:**
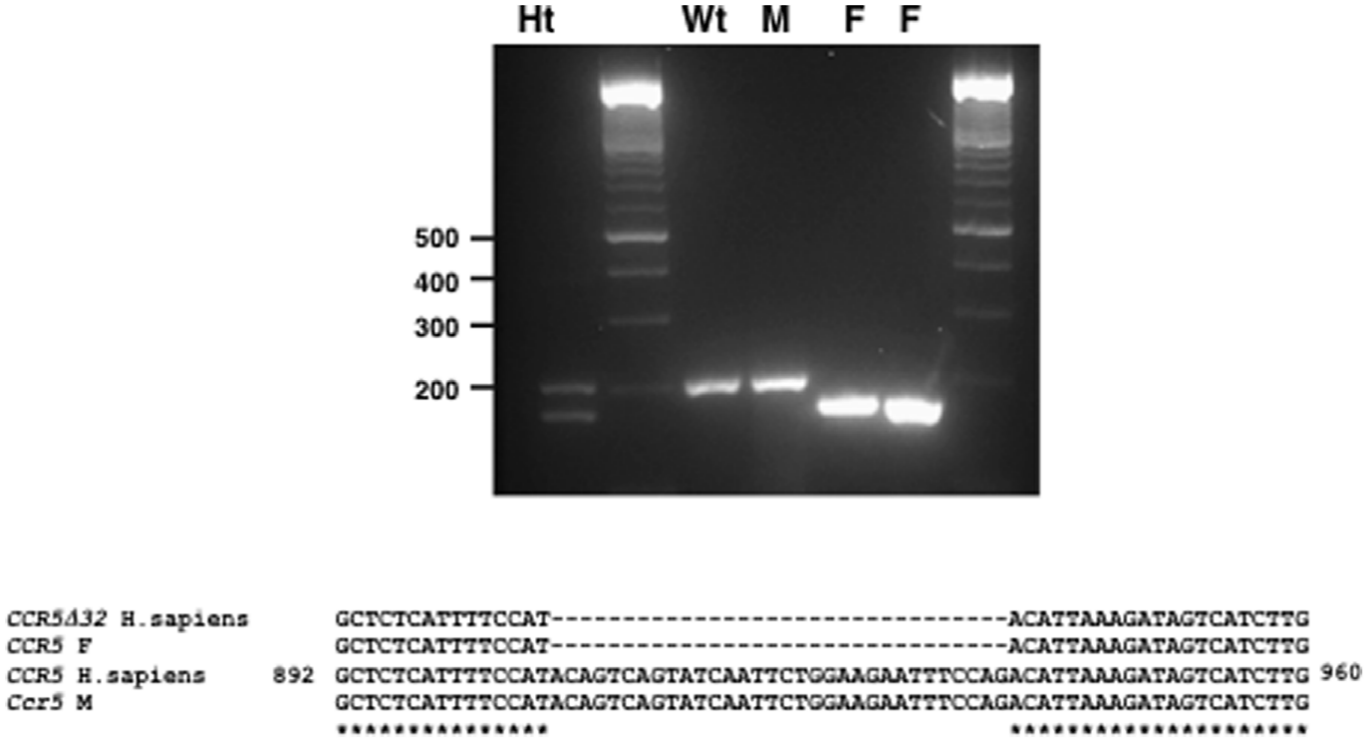
*CCR5* allele determination. Samples of bronchoalveolar lavages of M and F infected patients were used to isolate total RNA that was used for RT-PCR reactions and DNA sequencing to detect the *CCR5* region that comprises the 32-bp deletion. (Top), agarose gels with the amplified fragments from a human heterozygous sample (Ht), a wild type homozygous sample (Wt), the M-infected patient (M) and two independent samples taken from the F-infected patient. (Bottom), sequence determination of the M and F-infected patients. Sequences present in the databases for the human wild type *CCR5* receptor allele (*CCR5* H. sapiens) or the deleted allele (*CCR5Δ32* H.sapiens), have been used for comparison.

The chemokine receptor *CCR5* is expressed on activated macrophages and plays an important role in the macrophage response to influenza virus infection since knock out mice for *Crc5* gene display increased mortality rates [37]. Moreover, critically ill patients infected with pandemic H1N1 virus showed a large proportion of heterozygosity for a deleted allele of *CCR5* that prevents the surface expression of the protein (*CCR5Δ32*) [15]. With this information we examined whether the patients from whom the M and F viruses were isolated displayed differences in the *CCR5* alleles. Bronchoalveolar lavages were used to isolate total nucleic acids that were used for RT-PCR reactions. RNA was amplified by using previously reported primers surrounding the 32-bp deletion in the *CCR5* gene [15]. Wild-type *CCR5* RNA results in a 197-bp product, but the Δ32 allele yields a 165-bp product. The amplified products were analyzed in agarose gels together with the amplification products obtained from previously characterized *CCR5* homozygous (wt) and heterozygous (ht) human samples. The results showed that the M virus-infected patient was homozygous for the wild type *CCR5* allele, but the F virus-infected patient was homozygous for the *CCR5Δ32* deletion (Fig. 10, top). These results were further confirmed by sequencing the amplification products (Fig. 10, bottom).

All data reported above indicated that F virus replicates faster than M virus in human A549 cells and is more pathogenic in mice. Since the *CCR5* receptor could play a role in virus pathogenicity we performed genotypic analysis of the mice used in this study, as well as the A549 cell line. Thus, RNA samples isolated from mice kidneys or A549 cells were used for RT-PCR reaction and further sequencing of the *Ccr5* region that comprises the 32-bp deletion using specific primers for the human or mouse genes. The results obtained verified that all samples were homozygous for the wild type *Ccr5* allele (Fig. S2). Therefore, the differences observed for F and M viruses in the *in vitro* replication rates (Fig. 2), the pro-inflammatory cytokines and chemokines production (Fig. 3) and the pathogenicity in mice (Fig. 4) are independent of *CCR5* and rely exclusively on sequence differences between the two viral genomes.

## Discussion

Although the 2009 H1N1 pandemic virus caused relatively mild symptoms in most cases, sporadic severe disease or even death of infected patients was observed. The molecular basis for the severity of certain H1N1 pandemic infections has been a subject of intense scrutiny, including the analysis of potential virus pathogenicity markers and host predisposition, as well as co-morbid conditions in the patients. Here we have compared the viruses from two contemporary pH1N1 infected patients with no known co-morbid conditions that one developed a clinically mild disease (M) and the other resulted in a fatal case (F).

### Cytokines in Influenza Virus Infection

Recognition and rapid clearance of pathogens by the innate immune system provides the first line of defense upon infection. However, pathological inflammation is produced when excessive activation of the innate immune system occurs. Several reports pointed out the role of an excessive cytokine response (known as a cytokine storm) as a key contributor to morbidity and mortality of highly virulent 1918 and avian H5N1 influenza virus infections [38], [39] as well as in severe cases of pH1N1 influenza virus infection [40]. Accordingly, high levels of expression of a significant number of cytokines have been described in fatal cases of pH1N1 influenza virus infection [41]. The profile of cytokine response in severe pH1N1 influenza virus infections revealed a hyperactivation of the proinflammatory cytokines IL-6, IL-8, MCP-1, MIP-1β, GM-CSF and TNRF-1 that was not apparent in milder pH1N1 infections [42], [43], [44], [45], [46]. In addition an increase in RANTES whose natural receptor is CCR5, has been described in the bronchoalveolar lavage of patients with severe pneumonia associated with pH1N1 infections [47]. Among these proinflammatory cytokines, IL-6 may play an important role mediating severe disease caused by influenza virus infection, since increased levels of this cytokine correlate with infection with high virulent H1N1 strain in ferrets [48], severe infections of macaques with 1918 influenza virus [38] and pH1N1 virus [28], a fatal human case of pH1N1 [49] and with severe clinical manifestations in infected patients [50].

Analysis of cytokines induction in infected human alveolar epithelial cells with the M and F viruses as well as the seasonal A/New/Caledonia/20/99 (NC) strain revealed that F virus infection induces earlier and higher levels of IL-6, IL-8, MCP-1, GM-CSF and RANTES than M or NC infections (Fig. 3). Besides the role described for IL-6, IL-8, MCP-1 and GM-CSF in patients with pH1N1 severe infections, the chemokine RANTES, a natural agonist of CCR5 seems to control the production of IL-6, IL-8, MCP-1 and IFNs in influenza virus-infected cultured alveolar epithelial cells [51]. Therefore, it is conceivable that high levels of these chemokines and pro-inflammatory cytokines would increment cellular recruitment to the lungs at earlier times, thus contributing to inflammation and tissue consolidation. Indeed, there was a moderate increase in the histopathological damage in F virus- versus M virus-infected mice (Fig. 6–7). Also, this rapid induction of pro-inflammatory cytokines and chemokines supports the notion that a deregulation of the host immune response in the early stages of the infection, might contribute to increase the severity associated with more virulent pH1N1 strains.

### Pandemic H1N1 Influenza Virus Infection *in vivo*

The comparison of the F and M viruses in the murine model showed that F virus was more pathogenic, as indicated by the morbidity and mortality rates observed in the F-infected mice. The pathogenicity of several pH1N1 viruses in mice has been studied previously. Mice infected with pH1N1 virus showed weight loss and high titers in the lung but in most studies more than 10^6^ PFU were required to reach a 50% lethal dose or even the mice survived at the highest dose used [32], [52], [53]. In a particular study, the characterization of several strains of pH1N1 viruses in mice indicated efficient replication in the lungs but not extrapulmonary virus spread and no lethality, suggesting that pandemic viruses display mild to moderate virulence when compared with highly pathogenic viruses such as the 1918 virus [54]. In contrast 50% of the mice infected at 10^6^ PFU with the F virus but not with the M virus died, indicating that F virus is more pathogenic than other pH1N1 viruses. Moreover, infectious virus was recovered in higher proportion of animals and remained at later days post-infection in the hearts of F virus-infected mice (Fig. 5). Efficient dissemination of F virus might have contributed also to increase the severity of the infection. Accordingly, viral particles have been found in the hearts of both domestic ducks infected with H5N1 highly pathogenic avian virus [55] and fatal cases of 2009 pandemic infected patients [56]. Extrapulmonary complications of influenza virus infection have been previously reported. Around 50% of patients without previous cardiac history have abnormal electrocardiogram findings [57] and a link has been recently reported between influenza infection and acute myocardial infarction [58] and fulminant myocarditis in children [59] and adults [60].

Individuals who died with a confirmed influenza pandemic H1N1 virus infection showed some degree of bronchiolar epithelial necrosis and desquamation, as well as a pattern of exudative diffuse alveolar damage [61], [62], [63]. The histopathological and immunohistochemical examination of M virus- and F virus-infected mice detected more aggressive interstitial pneumonia or diffuse alveolar damage in the lungs of the F virus-infected mice, together with lung positive cells for NP antigen at late times post-infection. The results are consistent with the histophatological findings in autopsies of pandemic H1N1 infected patients that showed diffuse alveolar damage, acute massive intra-alveolar edema, neutrophilic bronchopneumonia and tracheobronchitis with some histopathological changes in alveoli [49], [62].

### Viral and Host Genetic Determinants

The genetic background of both the virus and the host are essential to determine the final outcome of an infection. Genetic determinants of influenza virus virulence have been mainly mapped to the polymerase genes (PB1, PB2 and PA), the hemagglutinin (HA), neuraminidase (NA), and non-structural protein 1 (NS1) [64]. In the case of pandemic H1N1 viruses, mutation D222G in the HA appeared as particularly relevant since it was significantly more frequent in severe cases of disease [65], [66], [67], [68]. Other reports described substitutions in the PB2 and PA polymerase genes [28] that were associated with severe disease and correlated with increased virulence in animal models [28], [69]. In other studies, no correlation between the clinical data and the virus replication capacity or virulence in mice could be observed [70] and no evidence was found that fatality rate could be attributed to specific virus changes in infected patients from Argentina [71]. Sequence comparison of M and F influenza viruses revealed 9 amino acid differences affecting the PB2 and PA polymerase subunits, the NP, HA and NA (Fig. 1). Besides the three conservative changes already described (aa 269 and 328 in PA and aa 400 in NP), aa 38K and 226K in HA and 274Y in NA that have been found in F virus are also present in the consensus sequence of contemporary pandemic viruses (Fig. 1), suggesting that these differences represent random drift or early virus adaptation to the new human host. However, mutations HA S127L, PB2 A221T and PA D529N have been observed very scarcely among the pH1N1 strains, human seasonal strains, avian and swine viruses (Table S2). In fact the total calculated frequency of appearance of HA S127L, PB2 A221T and PA D529N changes in the influenza viruses present in these host is 0.069%, 0.19% and 0.048%, respectively (Table S2) analyzed using the influenza virus resource databases from NCBI. On the other hand, no data exist either in the literature or in the data bases, reporting the sequence of any influenza A virus containing HA 127L, PB2 221T and PA 529N simultaneously, indicating that F virus is very infrequent and could have been generated and/or selected in the patient. These PB2 and PA residues are not present in any previously described functional motives of the polymerase subunits. On the other hand, HA 127 residue has not been previously involved in the structure of the pH1N1 HA antigenic sites or the receptor-binding pocket [72]. We are presently assessing the potential role of these amino acids as virulence markers.

Potential influenza virus host genetic determinants have been described and a significant increase on heterozygosis of a *CCR5* allele that contains a 32 bp deletion (*CCR5Δ32*) has been correlated with more severe course of the pH1N1 infection in a small cohort [15]. Recently, we have performed a screening with more than 200 samples, examining the presence of this mutation among Spanish patients with confirmed diagnosis of pH1N1 infection and its correlation with the severity of illness. Using a chi-square with Yates’ correction a significant increase in the proportion of fatal cases within patients with the *CCR5Δ32* mutation has been found (M. Cuevas et al. submitted). Around 15–20% of the Caucasian population is heterozygous for the *CCR5Δ32* allele, whereas the proportion of *CCR5Δ32* homozygous is below 1% [10]. The homozygous deletion results in failure to express the receptor on the cell surface and therefore a total inactive *CCR5* receptor. Influenza virus infected *Ccr5* knock-out mice display an accelerated macrophage accumulation in the lungs that is likely to be linked to enhanced expression of MCP-1 and RANTES and increased mortality rates associated with acute, severe pneumonitis [37]. Although a profounder characterization of the consequences of *CCR5Δ32* homozygosis for influenza virus infection is required, our data with those of other laboratories support a *CCR5* role in the course of influenza virus infection. Other potential influenza virus genetic determinants have been recently described as an increase on a single nucleotide polymorphism allele of the tumor necrosis factor [73] or an enrichment of a minor interferon-inducible transmembrane (IFITM) protein allele [74].

In summary, the F (A/CastillaLaMancha/RR5911/2009) virus replicates and induces cytokines in human alveolar epithelial cells very fast, including the pro-inflammatory IL-6 and IL-8 cytokines and the chemokine RANTES that seem to play an important role in influenza virus infection. In the murine model *in vivo,* the F virus showed a stronger morbidity and mortality than M virus and moreover higher proportion of mice having viral particles in the hearts of F-virus infected mice was found. Therefore the F patient was infected with a potential highly pathogenic virus and its genetic background of *CCR5Δ32* homozygous could contribute to the severity of the infection.

## Supporting Information

Figure S1 Profile of cytokines induction on A549 infected cells at 24 hpi. Cultured cells were infected at 3 PFU/cell with the A/New Caledonia/20/99 (NC) and the H1N1 pandemic M and F isolates. At 24 hpi cell supernatants were taken and used to determine the concentration of the indicated cytokines using the Luminex 100 System. Error bars indicate the standard deviation of the mean. Student’s *t*-test was performed to determine the *P* value. *P<0.05, **P<0.01, ***P<0.001,
(PDF)

Figure S2 *Ccr5* allele determination. Samples of cultured A549 (top) and kidneys from infected or uninfected mice (bottom)were used to isolate total RNA that was used for RT-PCR reactions and DNA sequencing to detect the *CCR5* region that comprises the 32-bp deletion. The sequence of the *CCR5* allele from human (*CCR5* H. sapiens) or mice (*Ccr5* M. musculus) have been used for comparison. The red line corresponds to the 32 bp deletion found in the *CCR5Δ32* samples.
(PDF)

Table S1 Nucleotides at the indicated positions of M and F RNAs.
(PDF)

Table S2 Frequency of different residues at PB2 221, PA 529 and HA 127 positions in human, swine and avian viruses.
(PDF)
