# Supplementary material for: Characterization In Vitro and In Vivo of a Pandemic H1N1 Influenza Virus from a Fatal Case
Source: PLoS One. 2013 Jan 10;8(1):e53515. doi: 10.1371/journal.pone.0053515 (PMC3542358; doi:10.1371/journal.pone.0053515)
Supplement: Figure S1 — Profile of cytokines induction on A549 infected cells at 24 hpi. Cultured cells were infected at 3 PFU/cell with the A/New Caledonia/20/99 (NC) and the H1N1 pandemic M and F isolates. At 24 hpi cell supernatants were taken and used to determine the concentration of the indicated cytokines using the Luminex 100 System. Error bars indicate the standard deviation of the mean. Student’s t-test was performed to determine the P value. *P<0.05, **P<0.01, ***P<0.001, (PDF) [file pone.0053515.s001.pdf]

Fig. Sup. 1

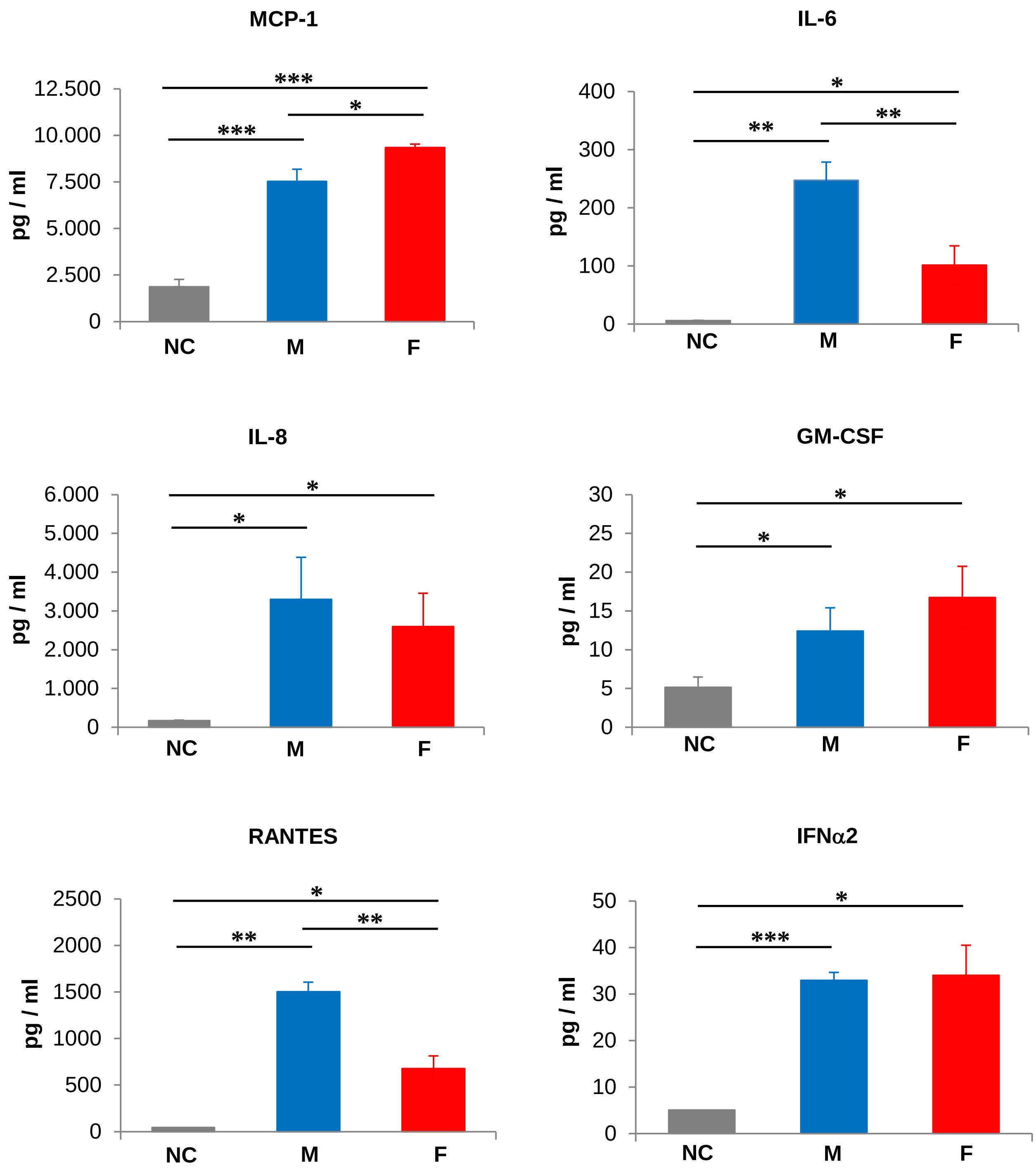

Fig. Sup. 1. Profile of cytokines induction on A549 infected cells at 24 hpi. Cultured cells were infected at 3 PFU/cell with the A/New Caledonia/20/99 (NC) and the H1N1 pandemic M and F isolates. At 24 hpi cell supernatants were taken and used to determine the concentration of the indicated cytokines using the Luminex 100 System. Error bars indicate the standard deviation of the mean. Student's *t*-test was performed to determine the *P* value. *$P < 0.05$, **$P < 0.01$, ***$P < 0.001$,.
