# Supplementary material for: Characterization In Vitro and In Vivo of a Pandemic H1N1 Influenza Virus from a Fatal Case
Source: PLoS One. 2013 Jan 10;8(1):e53515. doi: 10.1371/journal.pone.0053515 (PMC3542358; doi:10.1371/journal.pone.0053515)
Supplement: Figure S2 — Ccr5 allele determination. Samples of cultured A549 (top) and kidneys from infected or uninfected mice (bottom)were used to isolate total RNA that was used for RT-PCR reactions and DNA sequencing to detect the CCR5 region that comprises the 32-bp deletion. The sequence of the CCR5 allele from human (CCR5 H. sapiens) or mice (Ccr5 M. musculus) have been used for comparison. The red line corresponds to the 32 bp deletion found in the CCR5Δ32 samples. (PDF) [file pone.0053515.s002.pdf]

Figure S2

```
ccr5 H.sapiens     892 GCTCTCATTTTCCATACAGTCAGTATCAATTCTGGAAGAATTTCCAGACATTAAAGATAGTCATCTTG 960
ccr5 A549              GCTCTCATTTTCCATACAGTCAGTATCAATTCTGGAAGAATTTCCAGACATTAAAGATAGTCATCTTG
                       ********************************************************************

ccr5 M. musculus   645 GTCCTCATTTTCCACACACTCAGTATCATTTCTGGAAGAGTTTCCAAACATTAAAGATGGTCATCTTG 713
ccr5 M-infected       GTCCTCATTTTCCACACACTCAGTATCATTTCTGGAAGAGTTTCCAAACATTAAAGATGGTCATCTTG
ccr5 F-infected       GTCCTCATTTTCCACACACTCAGTATCATTTCTGGAAGAGTTTCCAAACATTAAAGATGGTCATCTTG
ccr5 MOCK-infected    GTCCTCATTTTCCACACACTCAGTATCATTTCTGGAAGAGTTTCCAAACATTAAAGATGGTCATCTTG
                       ********************************************************************
```

Figure S2. ccr5 allele determination. Samples of cultured A549 (top) and kidneys from infected or uninfected mice (bottom) were used to isolate total RNA that was used for RT-PCR reactions and DNA sequencing to detect the ccr5 region that comprises the 32-bp deletion. The sequence of the ccr5 allele from human (ccr5 H. sapiens) or mice (ccr5 M. musculus) have been used for comparison. The red line corresponds to the 32 bp deletion found in the ccr5Δ32 samples.
