# Supplementary material for: Characterization In Vitro and In Vivo of a Pandemic H1N1 Influenza Virus from a Fatal Case
Source: PLoS One. 2013 Jan 10;8(1):e53515. doi: 10.1371/journal.pone.0053515 (PMC3542358; doi:10.1371/journal.pone.0053515)
Supplement: Table S1 — Nucleotides at the indicated positions of M and F RNAs. (PDF) [file pone.0053515.s003.pdf]

Table S1. Nucleotides at the indicated positions of M and F RNAs

			M Virus				F Virus			
Segment	AA	nc POSITION	AA	nc	COV	%	AA	nc	COV	%
PB2	221	687	221A	G	900	99.67	221T	A	788	98.10
		688		C	897	99.78		C	787	99.11
		689		C	953	99.58		C	829	99.40
PA	269	831	269K	A	506	99.60	269R	A	563	99.64
		832		A	496	99.80		G	560	99.64
		833		A	505	100.00		A	549	99.45
PA	328	1008	328K	A	758	100.00	328R	A	782	99.74
		1009		A	757	100.00		G	778	99.23
		1010		A	712	99.72		A	719	99.86
PA	529	1611	529D	G	976	99.80	529N	A	961	98.96
		1612		A	987	97.06		A	997	97.99
		1613		C	1084	100.00		C	1206	98.92
NP	400	1224	400K	A	1171	99.83	400R	A	789	96.83
		1225		A	1170	99.40		G	793	99.24
		1226		G	1175	99.74		G	793	100.00
HA	38	138	38E	G	2237	99.91	38K	A	1074	99.44
		139		A	2180	99.86		A	1066	99.72
		140		A	2169	99.95		A	1058	99.91
HA	127	405	127S	T	657	99.85	127L	T	615	99.67
		406		C	645	100.00		T	599	98.83
		407		A	644	99.84		A	583	100.00
HA	226	702	226N	A	635	99.69	226K	A	420	100.00
		703		A	556	99.82		A	397	100.00
		704		T	592	64.70		A	403	98.76
NA	274	846	274F	T	1399	100.00	274Y	T	777	100.00
		847		T	1420	99.72		A	812	9.63
		848		T	1464	99.93		T	838	99.40

nc: nucleotide. AA: amino acid number. COV: number of times that the corresponding nucleotide has been read in total RNA. %: percentage of the corresponding nucleotide found in the total RNA samples.
Nucleotide differences between M or F viruses are represented in blue or red, respectively.
