# Supplementary material for: Characterization In Vitro and In Vivo of a Pandemic H1N1 Influenza Virus from a Fatal Case
Source: PLoS One. 2013 Jan 10;8(1):e53515. doi: 10.1371/journal.pone.0053515 (PMC3542358; doi:10.1371/journal.pone.0053515)
Supplement: Table S2 — Frequency of different residues at PB2 221, PA 529 and HA 127 positions in human, swine and avian viruses. (PDF) [file pone.0053515.s004.pdf]

Table S2. Frequency of different residues at PB2 221, PA 529 and HA 127 positions in human, swine and avian viruses

Subtype	PB2 221	Frequency (%)	Subtype	PA 529	Frequency (%)	Subtype	HA 127	Frequency (%)
H1N1 (Human)	04/01/2009-12/31/2011 (3028 sec.)		H1N1 (Human	04/01/2009-12/31/2011 (3118 sec.)		H1N1 (Human	04/01/2009-12/31/2011 (5549 sec.)	
	S (8)	0.26		E (1)	0.032		L (6)	0.108
	T (3)	0.099		N (1)	0.032		S (5543)	99.89
	A (3017)	99.6		D (2732)	99.9			
	01/01/1940-04/01/2009 (1111 sec.)			01/01/1940-04/01/2009 (1165 sec.)			01/01/1940-04/01/2009 (1675 sec.)	
	T (3)	0.27		N (1)	0,085		A (1)	0.059
	A (1108)	99.7		D (1164)	99.9		S (1574)	99.94
H3N2 (Human)	1940-2011 (2109 sec.)		H3N2 (Human)	1940-2011 (2739 sec.)				
	S (81)	3.84		D (2739)	100			
	T (1)	0.047						
	V (1)	0.047						
	A (2026)	96						
Any (Swine)	1940-2011 (816 sec.)		Any (Swine)	1940-2011 (904 sec.)		H1NAny (Swine)	1940-2011 (1245 sec.)	
	S (9)	1.1		N (2)	0.22		A (1)	0.08
	T (4)	0.49		D (890)	99.78		S (1244)	99.9
	V (5)	0.61						
	D (2)	0.24						
	A (796)	97.5						
Any (Avian)	1940-2011 (2526 sec.)		Any (Avian)	1940-2011 (2429 sec.)		H1NAny (Avian)	1940-2011 (196 sec.)	
	S (38)	1.5		N (1)	0.04		S (196)	100
	T (8)	0.36		D (2428)	99.96			
	V 5)	0.19						
	PB2 221	Total Frequency		PA 529	Total Frequency		HA 127	Total Frequency
	1940-2011 (9590 sec)			1940-2011 (10355 sec.)			1940-2011 (8665 sec.)	
	Any residue different than A	1.75		Any residue different than D	0.068		Any residue different than S	0.092
	T	0.19		N	0.048		L	0.069
